# Enhanced Room Temperature Ammonia Gas Sensing Properties of Fe-Doped MoO_3_ Thin Films Fabricated Using Nebulizer Spray Pyrolysis

**DOI:** 10.3390/nano12162797

**Published:** 2022-08-15

**Authors:** Fatemah H. Alkallas, Amira Ben Gouider Trabelsi, Mohd Shkir, Salem AlFaify

**Affiliations:** 1 Department of Physics, College of Science, Princess Nourah bint Abdulrahman University, P.O. Box 84428, Riyadh 11671, Saudi Arabia; 2 Advanced Functional Materials & Optoelectronic Laboratory (AFMOL), Department of Physics, Faculty of Science, King Khalid University, Abha 61413, Saudi Arabia; 3 Department of Chemistry and University Centre for Research & Development, Chandigarh, University, Mohali 140413, Punjab, India

**Keywords:** gas sensor, NSP, ammonia sensor, responsivity, reproducibility

## Abstract

MoO_3_ thin films are fabricated using nebulizer spray pyrolysis technique, which is doped with Fe at various concentrations of 1, 2, 3, and 4% for ammonia gas sensors application at room temperature. X-ray diffraction (XRD) study confirms the growth of the crystal by Fe doping up to 3%, nano rods shape morphology of the thin film samples observed by field emission scanning electron microscope (FESEM), reduction in bandgap is evidenced via UV-VIS spectrophotometer. Gas sensing study is performed using gas analyzing chamber attached with Keithley source meter. Since 3% Fe doped MoO_3_ sample displayed nano rods over the film surface which exhibits highest sensitivity of 38,500%, in a short period of raise and decay time 54 and 6 s. Our findings confirms that the 3% Fe doped MoO_3_ films suitability for ammonia gas sensing application.

## 1. Introduction

In today’s world, continuous detection of various gases is required for industrial process monitoring, automotive gas emission, and outdoor air quality monitoring [1]. Conventionally metal oxide semiconductors like SnO_2_, TiO_2_, WO_3_, MoO_3_, CeO_2_, Nb_2_O_5_, ZnO, etc. have been most frequently used as sensing materials [2,3,4,5,6,7,8,9,10]. The detection ability, operating temperature, and fabrication efficiency are the key factors that limit the real-time use of these gas sensors. Global technological efforts have been made to improve sensor selectivity, sensitivity, stability, repeatability, responsiveness, and recovery times [11,12,13,14,15]. Several materials have been developed so far, including molybdenum-based gas sensors such as molybdenum oxide (MoO_3_) and molybdenum sulphide (MoS_2_), allowing more gaseous chemicals adsorbed on its surface, resulting in high sensitivity [16,17,18,19,20,21,22,23]. MoO_3_ is an n-type semiconductor with a band gap between 2.39-2.9 eV which exhibits orthorhombic (α-MoO_3_), monoclinic (β-MoO_3_), hexagonal (*h*-MoO_3_), and ε-MoO_3_ phases [24,25,26]. Moreover, MoO_3_ suffers from disadvantages while satisfying the practical requirements like lower response and interference of various gases [27,28]. Currently, different strategies related to the surface modification, doping, nanoscale size and shape control are employed to ameliorate MoO_3_ gas sensing capability. Doping is one of these effective techniques optimizing the optical and electrical properties of MoO_3_ gas sensing. The ionic radius of Fe element is different than Mo element it generates some lattice deformation and oxygen vacancy formation which improve the gas response [29,30,31]. Fabrication of thin films using suitable technique is also important for producing suitable sensor material. Among different thin film coating techniques, nebulizer spray pyrolysis (NSP) offers various advantages in order to prepare homogeneous thin films [32]. Comparatively, NSP is a low-cost, eco-friendly and easy technique that can construct thin films with high growth rate in a non-vacuum atmosphere as well as it can deposit nanostructured material on the large glass substrate. Few previous studies have reported metal oxide thin film preparation using NSP and related techniques [33,34]. In this work MoO_3_:Fe thin films were fabricated using NSP technique, and its sensitivity towards the ammonia (NH_3_) gas is tested.

## 2. Materials and Methodology

Fe-doped MoO_3_ thin films were prepared by nebulizer spray pyrolysis method. In a typical thin film coating process, 0.005 M of ammonium heptamolybdate tetrahydrate ((NH_4_)_6_Mo_7_O_24_·4H_2_O) and a certain amount of Fe(NO_3_)_3_·9H_2_O (0, 1, 2, 3 and 4 wt.%) were dissolved in deionized water and stirred for 10 min. The as-prepared precursor was coated on glass substrate at temperature of 450 °C. The distance between substrate and spray gun maintained at 7 mm and the rate of flow of precursor solution is 1 mL/min. The coated films were kept at the coating temperature for 30 min. The obtained Fe-doped MoO_3_ thin films were subjected to know structural, optical, electrical and gas sensing properties. The micro structural spectacles of the fabricated samples were studied using a Pan-analytical X’Pert PRO X-ray diffraction (XRD) system. The surface morphology and surface coarseness were determined by field emission scanning electron microscopy (FESEM). A UV–vis–NIR spectrophotometer (Perkin Elmer Lambda, Waltham, MA, USA) was used to determine the transmittance, absorption spectra. A PerkinElmer LS55 fluorescence spectrophotometer with an excitation wavelength of 325 nm was utilized to detect the photoluminescence spectra at room temperature. A Keithley Source Meter (model 2450) was used to sense ammonia gas at ambient temperature (27 °C). The electrical current of the as-prepared thin films was obtained in both air and ammonia atmosphere.

### Gas Sensing Set Up

The Fe-doped MoO_3_ films were prepared, and the concentration of ammonia can be obtained using formula given in Equation (1) [25].

(1) $$C_{\left(\mathrm{ppm}\right)}=\frac{\delta \mathrm{VRT}}{{\mathrm{MP}}_{b}V_{b}}\times 10^{6}$$

Here, δ represents the testing gas density (g mL^−1^), T is the temperature of the sensor (K), R is the universal gas constant (8.415 J mol^−1^ K^−1^), P_b_ is the pressure in the chamber, V_b_ is the chamber volume in liters, V is the volume of the infused gas (mL), M specifies the molecular weight of the test gas (g mol^−1^). Here the sensor which is to be tested by placing the films inside the chamber.

Then, an adequate amount of ammonia was taken in to the chamber through a metallic beaker which is connected to a heating coil and a thermocouple was used to detect the temperature in the chamber. The ammonia vapor was produced by heating the ammonia solution up to the necessary temperature. As a result, the films began to sense the ammonia at room temperature, and the response current was recorded using a Keithley Source Meter. Once the response current reached to saturation condition, the chamber holding the films was opened and exposed to the atmospheric air. The procedure was continued for a range of NH_3_ concentrations. The metal contacts to the films are prepared using the silver (Ag) paste over the films surface for measuring the current response for all the variations in NH_3_ concentration. Finally, the overall current response towards the analyte gas was measured from the ratio of current in air (I_a_) condition and the current in the test gas (I_g_) condition.

## 3. Result and Discussion

### 3.1. XRD Analysis

**Figure 1:**
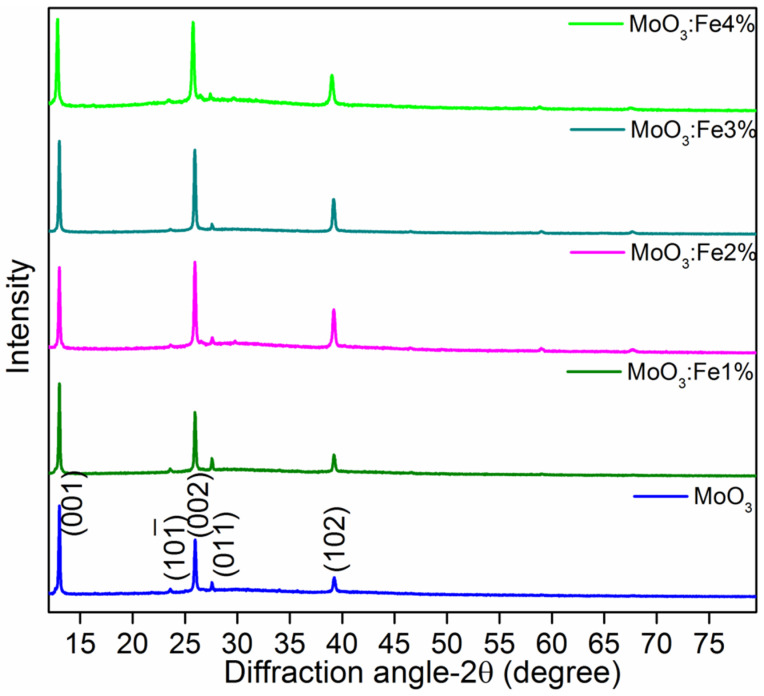
XRD patterns of Fe doped MoO_3_ thin films.

Figure 1, shows the XRD pattern of MoO_3_:Fe (0, 1, 2, 3 and 4%) thin films deposited using the nebulizer spray pyrolysis technique. It is noticeable from the figure, that all films display sharp peaks matched to the JCPDS card data 47–1320, of monoclinic structure [35]. It was clearly observed that the intensity of all the peaks of MoO_3_ varied due to the inclusion of Fe dopant, moreover the intensity of (002) plane increases with the increment in concentration of Fe dopants. This indicates that the crystallinity increases with respect to the incorporation of Fe dopants in MoO_3_. However, the further rise in the Fe dopants beyond the optimal level i.e., at 4% associated with a slight decline in the intensity of the (002) plane which might be originated by the domination of Fe ions in the target material MoO_3_ and the increase of lattice distortion and strain in MoO_3_.

The average crystallite sizes are calculated via the Debye Scherrer formula and the strain ε was calculated using Equations (2) and (3), respectively.

(2) $$D\;=\;\frac{0.9\;\lambda }{\beta Cos\theta }\;$$

(3) $$\varepsilon =\frac{\beta \;Cot\;\theta }{4}$$

where β depicts the full width half maximum (FWHM), *λ* denotes the wavelength of X-ray and *θ* is Bragg’s angle.

In Table 1, the crystallite size of MoO_3_:Fe (0, 1, 2, 3 and 4%) are shown, in which the crystallite size augments from 70 to 82 nm and greater concentration of Fe ion on the MoO_3_ matrix decreases to the size of 75 nm. This might be due to substitution of higher concentration of Fe atoms integrated in to the MoO_3_ lattice which distresses the crystallite size with the generation of large number of nucleation centers and increase in the lattice strain of the fabricated thin films [36]. Lattice constant values changed due to Fe doping and cell volume of the MoO_3_ sample increases up to 3% of Fe doping owning to the higher ionic radius of Fe^2+^ compared with host element of Mo^6+^.

### 3.2. Surface Morphologyanalysis

**Figure 2:**
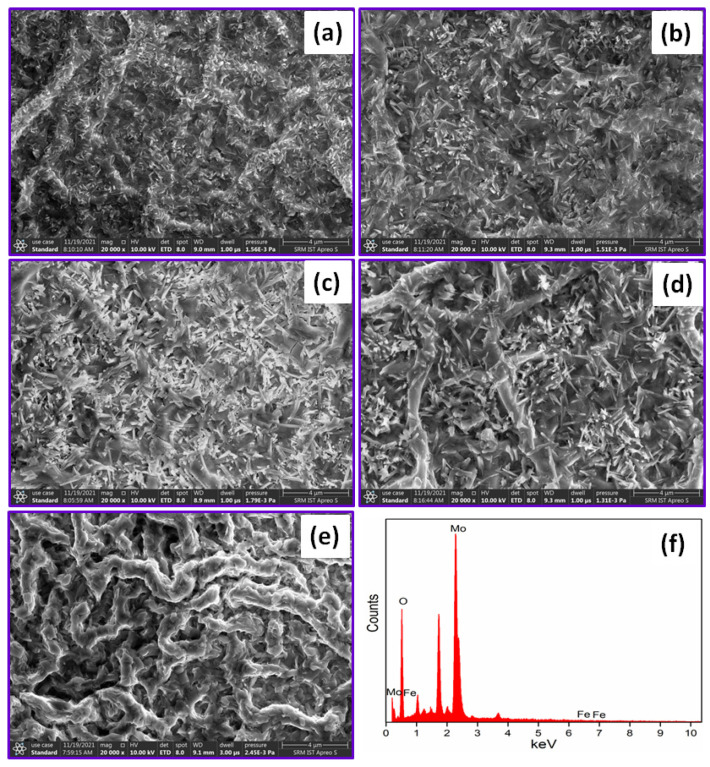
FESEM images of Fe doped MoO_3_ thin films (**a**) 0%, (**b**) 1%, (**c**) 2%, (**d**) 3%, (**e**) 4% of Fe and (**f**) EDX image of MoO_3_:Fe (3%) thin film.

The MoO_3_ nanostructures possess larger surface area, so they can exposed to absorb more gases, and the porosity of the material surface is favorable to support the gas diffusion, thereby enhancing the exploitation of sensing mechanism, and eventually improving its sensitivity [37]. Figure 2a–e, shows the nanobelts morphology with some nano rods over the MoO_3_:Fe (0, 1, 2, 3 and 4%) thin films. In 3% Fe doped MoO_3_ film has nano rods with porosity which is clearly visible in Figure 2d. Further increase in the dopant concentration i.e., Fe (4%), the high porosity gets decreased, which would reduce the gas sensing response. Figure 2f, shows EDX pattern of as prepared thin film, which confirms the presence of Mo, Fe and O in the final deposited film.

### 3.3. Optical Property Analysis

**Figure 3:**
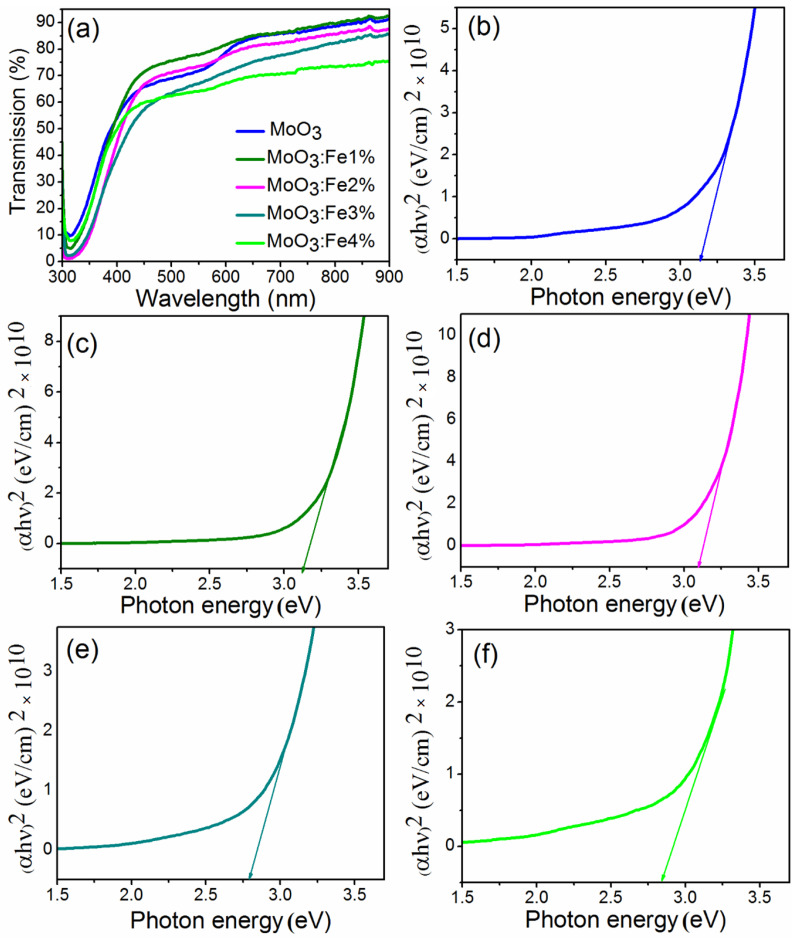
(**a**) Transmission spectra and Tauc’s plot of Fe doped MoO_3_ thin films (**b**) 0%, (**c**) 1%, (**d**) 2% (**e**) 3%, (**f**) 4% of Fe concentrations.

Figure 3 represents the transmission spectra of MoO_3_:Fe (0, 1, 2, 3 and 4%) thin films observed within the wavelength range 300–900 nm. It has been noticed that the transparency of films increases with the increase of dopant concentration and reaches maximum transparency for MoO_3_:Fe (3%) due to the enlarged voids generated in the thin films, which can be identified through the SEM image. This is ascribed to the structural deformations occurred by the amalgamation of Fe^2+^ ions which substitutes the Mo^6+^ ions from the host MoO_3_ matrix [38]. The diminution in transmittance for Fe dopants (1, 2, and 4%) are due to the amplified scattering of photons originated by the crystal defects, where the voids are less compared to the Fe (3%) dopant concentration [39,40]. 

Figure 3 illustrates the Tauc’s plot for obtaining the optical band gap (*E_g_*) of the fabricated thin films MoO_3_:Fe (0, 1, 2, 3, and 4%) calculated using Equation (4) [41].

(4) $$\alpha hv^{2}=A(hv-E_{g})$$

where *hν* denotes the photon energy, A indicates a constant for a direct transition, and α represents the optical absorption coefficient, the energy gap of the developed thin films was obtained through *hv* vs. (*αhv*)^2^ plot by extending the linear segment of the curve to the energy axis. The optical band gap of MoO_3_:Fe doped thin film shows decreasing from 3.13 to 2.79 eV for the doped concentration Fe (0%) to Fe (3%) dopant ions on the MoO_3_ matrix. On further rise of doping concentration from Fe (3%) to Fe (4%) the band gap of MoO_3_ tends to increase to 2.82 eV which was similar to the earlier reported values [42]. The increase in carrier concentration causes the narrowing of band gap as the concentration of Fe doping increases, which is related to the raise in film defects. According to the Burstein–Moss effect, as the carrier concentration increases from Fe (0 to 4%), the lowest energy state of the conduction band is filled with the electrons, thus a red shift is observed in the Fermi level while doping from Fe (0%) to Fe (3%), and a blue shift is observed for Fe (4%) film, due to the increase in band gap, as a result of the heavily doped Fe^2+^ ion on the host MoO_3_ [40,41,42,43].

### 3.4. Photoluminescence Analysis

**Figure 4:**
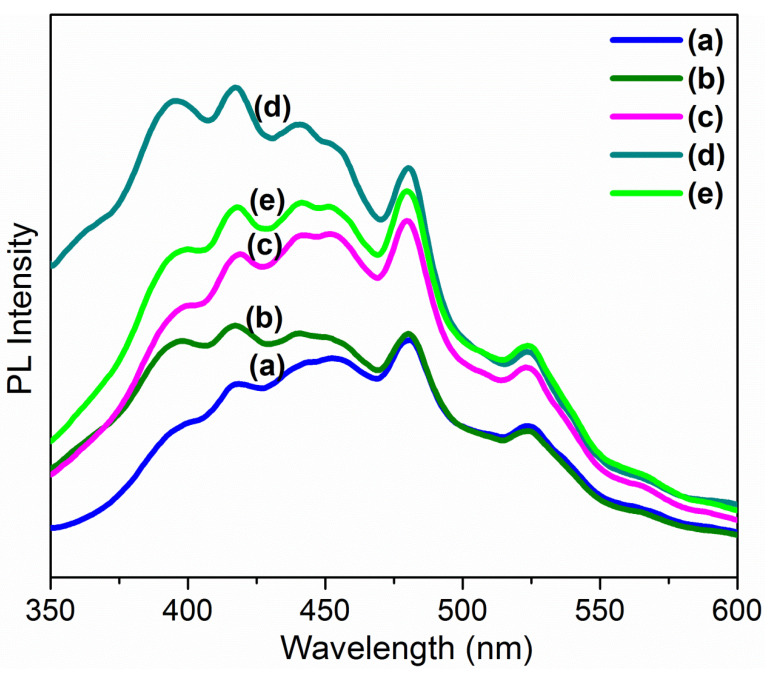
PL spectra of Fe doped MoO_3_ thin films. (**a**) 0%, (**b**) 1%, (**c**) 2%, (**d**) 3%, (**e**) 4% of Fe concentrations.

Figure 4 represents the photoluminescence spectra, obtained at room temperature within the wavelength range of 300 to 600 nm. It is well known that all the emission peaks like 396, 416, 441, 453, 480, 523 nm corresponds to transitions among the diverse intrinsic defect states within the band gap of MoO_3_:Fe thin films [44]. Nevertheless, the change in morphology and crystallite size caused by Fe doping has a noteworthy effect on the emissivity of as-prepared thin films. It can be clarified that the broad PL spectra shows the event of UV emission and visible light emission from MnO_3_:Fe film. Here the PL spectra exhibit two emission bands such as UV (389 nm) and visible [Violet (416 nm), blue (441 nm) and bluish green (453 nm and 480 nm) green (523 nm)]. The UV emissions are due to the well-built near-band edge emission (NBE) owing to the free-exciton recombination in the fabricated thin films and, similarly visible light emissions are owed to the changeover of excited optical centers into the deep levels. These deep level emissions are due to the existence of impurities as dopants in the host MoO_3_, along with the surface defects, and its potency is positively allied with the defect density. Thus, in the as-prepared thin films shows the enhanced crystallinity leads to an increase in the strength of band-edge emission [42]. The emission peaks such as 389, 416, 441, 453 and 480 nm bands of Fe (3%) shows red shift towards a longer wavelength, similarly a blue shift is observed for the higher dopant concentrations towards the shorter wavelength compared to Fe (0%) is due to the quantum confinement, followed by the widening of band gap with the decrease in particle size with respect to the dopant concentration. The green emission was observed at 523 nm, which is ascribed to the transitions sandwiched between the electrons close to the conduction band and the deep spellbound holes which are due to the ionized oxygen vacancies. This green emission is also credited to doubly charged oxygen vacancy centers situated at the surface of the thin film [45]. The intensity of Fe (3%) of NBE is higher than the visible deep level states, due to the fact that surface defects, lattice defects and stoichiometric ratio of as-prepared thin films, which generates more oxygen vacancies in the host material. The improvement of PL intensity in case of Fe (3%) dopant concentration is due the rise of porosity and roughness due ti increase in the surface areathereby producing an energetic response towards the incoming ultraviolet radiation. On other hand the smooth surfaced thin films shows specular reflection, but a coarse nature of thin film will permit the excitation light to be reflected onto a different segment of the film surface, hence it results highly well-organized utilization of the excitation energy and consequently possess a higher PL intensity [42].

### 3.5. Gas Sensing 

**Figure 5:**
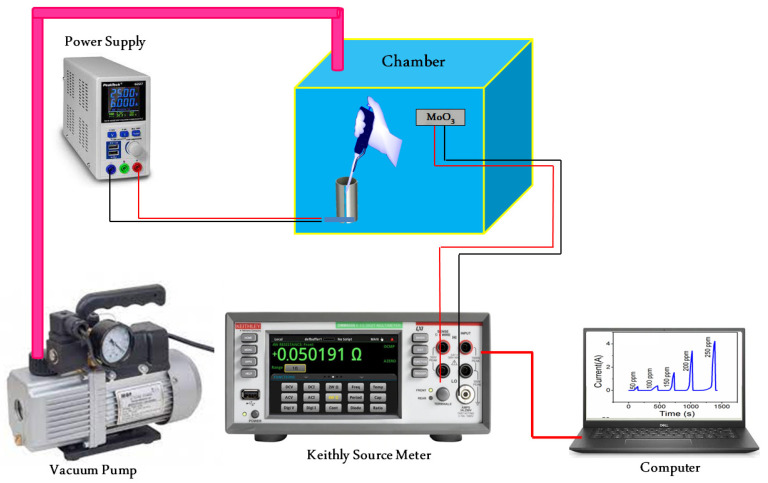
Schematic diagram of gas sensing setup for ammonia gas detection.

In wide range of semiconducting materials, the electrical spectacles of thin films may change when definite amount of gas molecules are adsorbed on its surface. Here the sensing properties of MoO_3_:Fe (0, 1, 2, 3 and 4%) thin films can be identified as the function of electrical current to the NH_3_ with different exposed on the film surface as mentioned in schematic Figure 5. Since the extent of sensing depends on the interaction between MoO_3_:Fe thin films and surrounding gases. Typically the sensing of NH_3_, gas molecules related to the surface coarseness, crystallite size, the absorptive nature of the surface, dopants concentration and surface defects of the as-prepared thin films affect the sensing response [37,46]. In the prior reports, the sensing efficiency of Fe doped MoO_3_ materials towards ammonia gases is higher due to the catalytic consequence of large surface area [37]. MoO_3_:Fe sensing behavior depends on the composition, crystallite size, surface morphology, which is enhanced by the impact of Fe dopants in MoO_3_ nanostructures [47,48]. In case of critical surface state density, the total carrier concentration within the grains, decreases with the crystallite size. Because of bigger grains observed in Fe (3%) on MoO_3_ thin films, increases the amount of charge carriers which is responsible for the conductivity mechanism, and the responsivity reaches 18,900% which is higher than the earlier reported values [49,50], along with the raise or ON state occurs at 51 s and decay or OFF state at 8 s.

**Figure 6:**
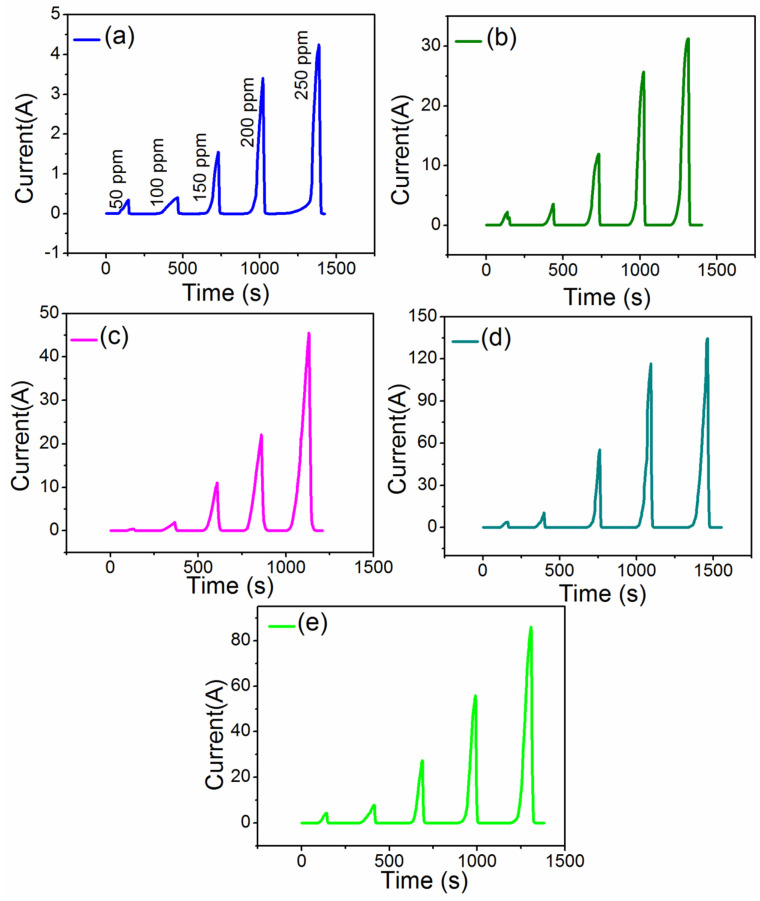
Time−Current of Fe doped MoO_3_ thin films (**a**) 0%, (**b**) 1%, (**c**) 2%,(**d**) 3% and (**e**) 4% of Fe concentrations.

From the Figure 6, it was observed higher response for Fe (3%) film than with other doped films of Fe (1%), Fe (2%), Fe (4%) and the previous reported values [51]. On other hand, it was noticed that for the certain number of surface state density, the net carrier concentration decreased in the case of 1, 2 and 4% Fe doped MoO_3_ film compared to the 3% Fe doped film. Figure 6 also shows that the electrical resistance of MoO_3_:Fe thin films varies as the function of ammonia (NH_3_) gas concentrations of 50–250 ppm at room temperature. The NH_3_ gas sensitivities are drastically increased, with the rise in concentration. The optimum NH_3_ concentration is found to be 250 ppm for the as- prepared thin films of MoO_3_:Fe (0, 1, 2, 3, and 4%). PL spectra revealed that MoO_3_:Fe (3%) film has more oxygen vacancies than the other films. Consequently, the doping process appreciably improves the gas sensing properties of MoO_3_:Fe nanostructures. 

**Figure 7:**
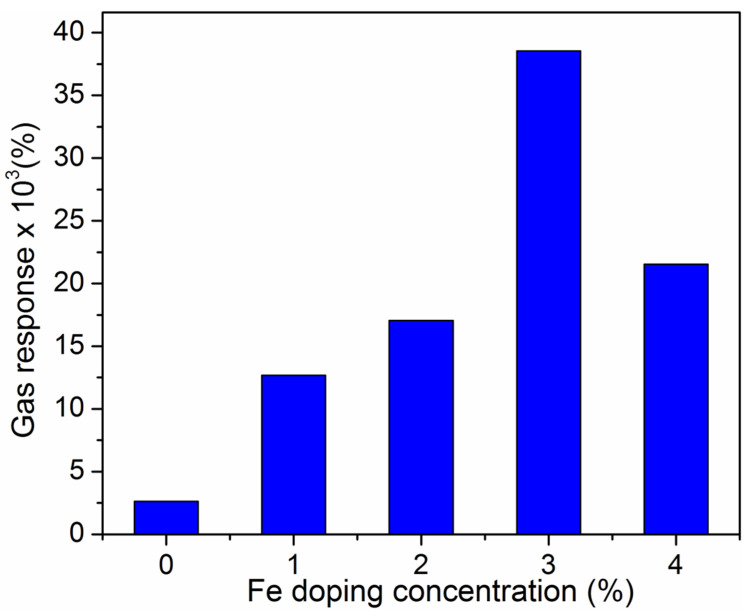
Gas response of Fe doped MoO_3_ thin films.

The film sensing response is calculated from the ratio of the electrical current of the film in air to that of the film exposed to the NH_3_ gas [52]:

(5) $$S\left(\%\right)=\frac{I_{g}-I_{a}}{I_{a}}$$

where *S*, *I_a_* and *I_g_* stands for the sensitivity, film current in air (darkness) and film current in the presence of NH_3_ gas. Figure 7, shows the disparity of sensing reaction with different NH_3_ gas concentrations for the as-prepared thin films. It can be seen from Table 2 that the response (%) for 250 ppm of maximum concentration of NH_3_ gas was 2620% for MoO_3_:Fe (0%), with response time of 62 s for raise or on state, and 8s decay or off state of gas sensor. This response (%) probably increased while adding dopants of about Fe (3%), resulting in maximum value of 38,500% with short period of raise and fall time of about 54 s and 6 s. In this case, for Fe (3%) on the MoO_3_ host reaches proper neutral charge vacancy, thereby creating high level of oxygen vacancies in the system, but for the other dopant concentrations like Fe (1, 2 and 4%), due to the mismatch of stochiometric ratio, proper oxygen vacancies are not allowed in to the host system, hence the Fe (3%) shows good sensing response than the other films with high response current [53]. This might be because of the better crystallinity, surface area, adsorption and defect sites sensors [54,55,56]. Herein, we have compared the current ammonia gas sensing results of MoO_3_ sample with other published work and the comparison table is displayed in Table 3. Andreas et al. [57] fabricated α-MoO_3_ sample by Si-doping via spray pyrolysis technique exhibited notable response and recovery time of 3.6 min/7 min for 400 ppm ammonia concentration. The MoO_3_ sample synthesized using sol–gel technique by Prasad et al. [58] displayed reasonable raise and decay speed of 15 s/20 s, but the sample operating temperature was 500 °C. Sunu et al. [59] readied Pulsed laser deposition of MoO_3_ thin films as ammonia sensor at 280 °C working temperature showed response and recovery speed of 15 s/300 s. Recently Neha et al. [60] and Chua et al. [61] manufactured 2D hexagonal MoO_3_ and h-MoO_3_ samples responded towards 5 ppm and 500 ppm ammonia gas limit at room temperature with the improved response and recovery speed of 69 s/129 s and 210 s/241 s respectively. The sample prepared in the present work i.e., Fe (3%) doped MoO_3_ sample displayed better ammonia gas response (250 ppm) at room temperature with smaller response and recovery time of 54 s/6 s.

**Figure 8:**
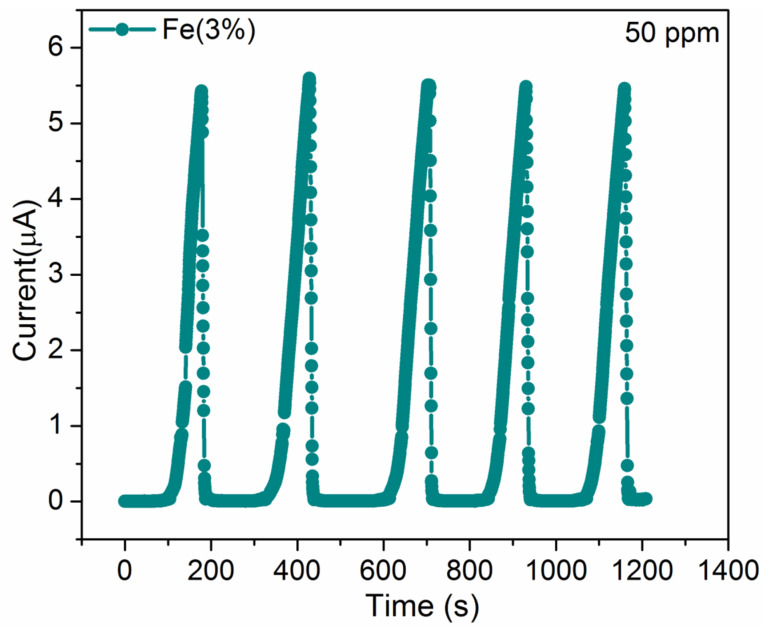
Cycle test of MoO_3_:Fe (3%) thin film.

The stability and the reproducibility preside over the device excellence. The film MoO_3_:Fe (3%), shows good sensing performance, therefore, it is investigated for its performance after 2 months. Importantly, the film was stored under atmospheric condition. Figure 8, shows the reproducibility of the sensing performance of MnO_3_:Fe (3%)film was used after a couple of months which is stored in laboratory conditions are checked for its performance stability, by taking 5 cycles each time for the maximum 250 ppm NH_3_. Therefore, it is clearly understood that the electrical current of the newly fabricated film and the old film shows identical behavior response as the freshly prepared thin film. It predicts that the NH_3_ gas molecules interaction with MoO_3_:Fe (3%) is not affected by any of the atmospheric condition. From this inspection, it is understood that the stability and lifetime of the MoO_3_:Fe (3%) prepared film shows better enhancement in the gas sensing application even after aging its stability remains constant.

Cross-sensitivity of the as-prepared sensors was checked by other gases and the gas response values of the fabricated films are 216, 364, 412 and 2089 for isopropanol, ethanol, methanol and ammonia. It is observed that the film shows maximum response to ammonia compared to isopropanol, ethanol, and methanol. Such a high response of ammonia could be assigned to its electron donating ability which is elevated compared to other tested gases due to the presence of lone pair of electrons.

Typically, a number of mechanisms can be well thought-out for the sensing response of MoO_3_ thin films with regards to ammonia gas. It is noteworthy to mention that the presence and enhancement of oxygen vacancies seems to be one of the major reasons which modifies the electrical properties of the as-prepared sensor. Since the oxygen could be simply adsorbed on the surface of the film, when exposed to gas or even heating at room temperature. The adsorbed oxygen on the surface of the film, when exposed to air leads to the abduction electrons tends to form O_2_^−^ and O^−^, which relies on the temperature of the film. O_2_^−^ is typically produced below 100 °C, whereas O^−^ is produced between 100 and 300 °C, and O_2_^−^ is produced beyond 300 °C [53]. These different species are produced when ambient oxygen chemically adsorbs to the film surface, eliminating the electrons that were previously present in the conduction band [54]. From the earlier, at room temperature, MoO_3_:Fe films with 0, 1, 2, 3, and 4% Fe content may have formed O_2_^−^ species. The chemisorbed oxygen increases the films current when it reacts to being exposed to incoming NH_3_ gas. MoO_3_ is an n-type semiconducting material with a wide bandgap, consequently a large number of oxygen can be adsorbed onto surface of thin films in atmospheric conditions. Furthermore, some of the precise type gases can interrelate greatly with the oxygen of MoO_3_ and thus alter the electrical response of the film towards the incoming gas. Since the defects created by the donor like oxygen vacancies and interstitials of Fe on MoO_3_ are the most constructive path for the oxygen species, and thus enhancing the probability of interface effect to the NH_3_ molecules. Besides, when the donor content increases, which lead to the formation of defects, more oxygen molecules get adsorbed the film surface and can respond to the NH_3_ gas, thus enriching the gas sensing response of the prepared thin film [55,56]. The interaction of gas with MoO_3_ is expressed by the following expression using Equations (6) and (7):

(6) $${\mathrm{NH}}_{3\left(\mathrm{ads}\right)}+O^{-}{}_{\left(\mathrm{ads}\right)}\to \;{{\mathrm{NH}}_{3}}^{-}{}_{\left(\mathrm{ads}\right)}$$

(7) $$2{\mathrm{NH}}_{3\left(\mathrm{ads}\right)}+3O^{-}\to N_{2\left(\mathrm{ads}\right)}+3H_{2}O+3e^{-}$$

Since the straight forward absorption and desorption of NH_3_ gas and the oxygen molecule are given by Equations (8) and (9).

(8) $${\mathrm{NH}}_{3\left(\mathrm{gas}\right)}\to {\mathrm{NH}}_{3\left(\mathrm{ads}\right)}$$

and

(9) $$O_{2\left(\mathrm{ads}\right)}\to O_{2\left(\mathrm{ads}\right)}$$

hence, the negatively charged O^−^ and O_2_^−^ are formed on the film surface, using Equation (10):

(10) $$O_{2\left(\mathrm{ads}\right)}^{-}+e\to 2O_{\left(\mathrm{ads}\right)}^{2}$$

**Figure 9:**
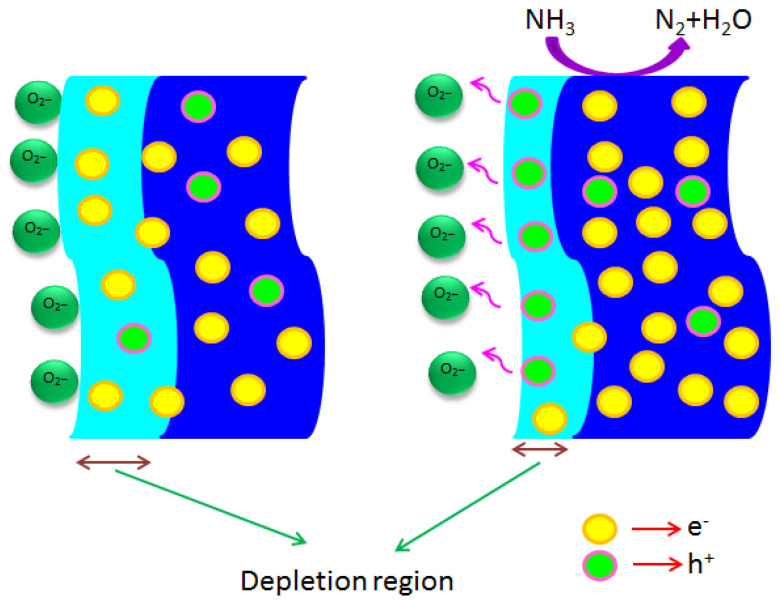
Schematic diagram of MoO_3_ gas sensing mechanism.

From the above metioned relations, it is noted that the discharge of electrons spellbound in the film surface, which causes the increase in the response current when NH_3_ molecules responds to the O^−^ species. The maximum gas response of the samples depends on two factors: the quantity of electrons trapped from the conduction band through the process of adsorption of oxygen species, and the quantity of electrons released by the adsorbed oxygen species by reaction with ammonia gas. These pathways allow for the complete release of all trapped electrons from the adsorbed oxygen species, demonstrating the critical function that ammonia gas can play in the exchange of electrons. When ammonia interacts with the MoO_3_:Fe film surface, the electron depletion region, grain boundaries, and Fermi energy (EF) are also reduced. The schematic ammonia gas sensing diagram of MoO_3_ film is shown in Figure 9. The speedy response to ammonia gas can also be attributed to the distribution of porous nanobelts with greater surface coarseness with the increased surface area while going towards the higher dopant concentration. On the other hand, the switch over of electrons in the Fe^2+^ ions with MoO_3_ are responsible for the decrease in the oxidation state, and thereby increasing the depletion region, resulting in the enhanced of the sensor response for MoO_3_:Fe (3%).

## 4. Conclusions

This work aims to prepare reproducible chemo resistive gas sensors made ofMoO_3_:Fe (0, 1, 2, 3 and 4%) thin film sensor. Here four different concentrations of Fe are used as a dopant on MoO_3_ matrix, which was successfully synthesized and confirmed the monoclinic phase of MoO_3_ thin films through XRD. FESEM analysis revealed that the 3 % Fe doped MoO_3_ film possess nano belt morphology with some porosity which enhances the gas sensing behaviour. The MoO_3_:Fe (3%) thin film shows better transparency and least bandgap value of 2.79 eV. PL shows visible, blue and green emission doubly charged oxygen vacancy centers situated at the surface of the as-prepared thin film, which makes it superior to enrich the gas sensing response. The gas sensing response of the MoO_3_:Fe (3%) thin film showed a drastically improved responsivity with short period of on/off response to the 50 ppm to 250 ppm of NH_3_ gas. Thus, MoO_3_:Fe (3%) thin film could be better suited as gas sensing material for room temperature ammonia sensing.

## Figures and Tables

**Table 1:** Structural parameters and lattice constant values of Fe doped MoO_3_ thin films with different Fe doping concentrations.

Fe Doping Concentration (%)	Crystallite Size (nm)	Strain ×10^−3^	Lattice Constants			Cell Volume (Å^3^)
			*a* (Å)	*b* (Å)	*c* (Å)	
0	70	4.40	3.968	3.684	7.060	100.09
1	72	4.24	3.971	3.686	7.073	100.39
2	78	3.90	3.950	3.684	7.165	100.94
3	82	3.75	3.943	3.691	7.225	101.63
4	75	4.11	3.944	3.691	7.237	101.81

**Table 2:** Gas sensing parameters of Fe doped MoO_3_ thin films with different Fe doping concentrations.

Fe Doping Concentration (%)	Response (%)	Rise Time (s)	Decay Time (s)
0	2620	62	8
1	12,700	60	7
2	17,100	60	7
3	38,500	54	6
4	21,500	59	9

**Table 3:** Comparison of ammonia sensing performances of Fe doped MoO_3_ thin films with other MoO_3_ based sensors.

Material	Operating Temperature	Concentration (ppm)	Response Time	Recovery Time	Ref.
Si doped MoO_3_	400 °C	400 ppm	3.6 min	7 min	[57]
MoO_3_	500 °C	-	15 s	20 s	[58]
MoO_3_	280 °C	500 ppm	15 s	300 s	[59]
MoO_3_	RT	5 ppm	69 s	129 s	[60]
MoO_3_	RT	500 ppm	210 s	241 s	[61]
Fe doped MoO_3_	RT	250 ppm	54 s	6 s	Present work

## Data Availability

The raw/processed data required to reproduce these findings cannot be shared at this time as the data also forms part of an ongoing study.
